# Neutrophil-to-lymphocyte ratio for the prognostic assessment of hepatocellular carcinoma: A systematic review and meta-analysis of observational studies

**DOI:** 10.18632/oncotarget.9942

**Published:** 2016-06-10

**Authors:** Xingshun Qi, Jianjun Li, Han Deng, Hongyu Li, Chunping Su, Xiaozhong Guo

**Affiliations:** ^1^ Department of Gastroenterology, General Hospital of Shenyang Military Area, Shenyang, Liaoning 110840, China; ^2^ Department of Radiotherapy, The First Affiliated Hospital, China Medical University, Shenyang, Liaoning 110001, China; ^3^ Library of Fourth Military Medical University, Xi'an, Shaanxi 710032 China

**Keywords:** hepatocellular carcinoma, inflammatory, neutrophil, lymphocyte, prognosis

## Abstract

**Background and aims:**

Neutrophil to lymphocyte ratio (NLR) is an inflammatory-based marker. A systematic review and meta-analysis was performed to explore the prognostic role of NLR in patients with hepatocellular carcinoma (HCC).

**Results:**

Overall, 598 papers were identified, of which 90 papers including 20,475 HCC patients were finally included. Low baseline NLR was significantly associated with better overall survival (HR = 1.80, 95% CI: 1.59–2.04, *p* < 0.00001) and recurrence-free or disease-free survival (HR = 2.23, 95% CI: 1.80–2.76, *p* < 0.00001). Low post- treatment NLR was significantly associated with better overall survival (HR = 1.90, 95% CI: 1.22–2.94, *p* = 0.004). Decreased NLR was significantly associated with overall survival (HR = 2.23, 95%CI: 1.83–2.72, *p* < 0.00001) and recurrence-free or disease-free survival (HR = 2.23, 95% CI: 1.83–2.72, *p* < 0.00001). The findings from most of subgroup meta-analyses were consistent with those from the overall meta-analyses.

**Materials and Methods:**

All relevant literatures were identified via PubMed, EMBASE, and Cochrane library databases. Hazard ratio (HR) with 95% confidence interval (95%CI) was calculated. Subgroup meta-analyses were performed according to the treatment options, NLR cut-off value ranges, and regions.

**Conclusions:**

NLR should be a major prognostic factor for HCC patients. NLR might be further incorporated into the prognostic model of HCC.

## INTRODUCTION

Prognostic assessment of hepatocellular carcinoma (HCC) is very important for clinicians and patients. The relevant knowledge is being rapidly accumulated. Traditional prognostic variables mainly include portal vein thrombosis, tumor size, and alpha-fetoprotein, etc. [1]. As for the prognostic staging of HCC, the Barcelona Clinic Liver Cancer (BCLC) system is the most frequently used tool with 5 major parameters, such as tumor size, tumor number, Child-Pugh class, physical status, and tumor metastasis [2]. Several alternative staging systems include the Cancer of the Liver Italian Program (CLIP) system [3], the Hong Kong Liver Cancer (HKLC) system [4], and the Japan Integrated Scoring (JIS) system [5]. As for the liver function assessment of HCC, Child-Pugh class is the most frequently used tool with 5 variables, such as bilirubin, albumin, international normalized ratio, ascites, and hepatic encephalopathy [6]. Albumin-bilirubin score is a recently developed and more convenient tool [7]. More recently, the associations of inflammation-based markers with the prognosis of HCC have been actively explored. Neutrophil to lymphocyte ratio (NLR), which refers to the ratio of neutrophil to lymphocyte count, is a readily available marker for assessing the systemic inflammatory changes. NLR reflects the potential balance between neutrophil-associated pro-tumor inflammation and lymphocyte-dependent anti-tumor immune function [8–11]. An elevated NLR may represent a trend towards increased pro-tumor inflammation and decreased anti-tumor immune function. Herein, we have conducted a systematic review and meta-analysis to analyze the prognostic role of NLR in HCC patients treated with different treatment options. This work was registered at PROSPERO database (registration number: CRD CRD42016033409).

## RESULTS

### Study selection and characteristics

**Figure 1:**
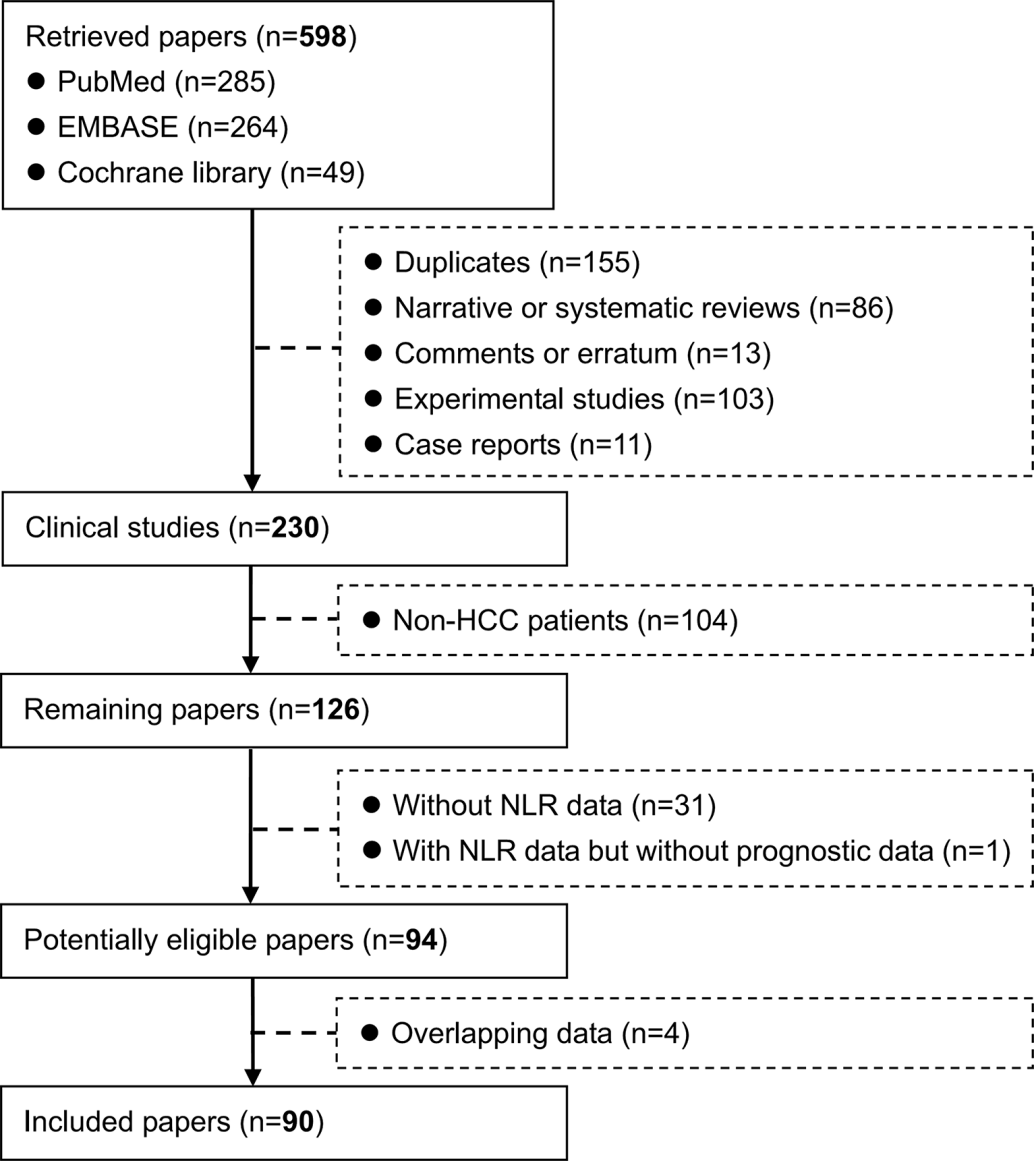
Flowchart of study inclusion

A total of 598 papers were identified. Among them, 90 papers with 20,475 HCC patients were included in the systematic review (Figure 1) [12–101]. Study characteristics were summarized in Table 1. According to the publication type, 21 and 69 papers were published in abstract and full-text forms, respectively. According to the study design, 60 and 5 papers were retrospective and prospective, respectively; 2 papers were both retrospective and prospective; and the study design was not available in 23 papers. According to the regions, 63, 14, and 13 studies were conducted by Asian, European, and American researchers, respectively.

**Table 1:** Study characteristics

First author	Journal (Year)	Type of publication	Study design	Regions	Enrollment period	Study population	No. Pts
Abdelmessih RM	Hepatology (2011)	Abstract	Retrospective	NY, US	1999.3– 2010.4	HCC patients who were downstaged with TACE prior to LT	200
Afshar M	Journal of Hepatology (2015)	Abstract	Retrospective	Birmingham, UK	2009.4– 2014.3	HCC patients treated with sorafenib	217
Agopian VG	Journal of the American College of Surgeons (2015)	Full-text	Retrospective	CA, US	1984– 2013	HCC patients treated with LT	865
Aino H	Molecular and Clinical Oncology (2014)	Full-text	NA	Fukuoka, Japan	1998.4– 2012.4	Advanced HCC patients with extrahepatic metastasis	419
Bertuzzo VR	Transplantation (2011)	Full-text	Retrospective	Bologna, Italy	1997– 2009	HCC patients treated with LT	219
Bodzin A	American Journal of Transplantation (2015)	Abstract	Retrospective	CA, US	1984– 2014	Recurrent HCC after LT	106
Bronson N	HPB (2012)	Abstract	Retrospective	PA, US	2002.6– 2011.7	HCC patients treated with resection	68
Bruixola G	Journal of Clinical Oncology (2015)	Abstract	Retrospective	Valencia, Spain	2008– 2014	HCC patients treated with sorafenib	145
Chan AW	Annals of Surgical Oncology (2011)	Full-text	Retrospective	Hong Kong, China	2001.1– 2011.12	BCLC stage 0/A primary HCC patients treated with surgical resection	597
Chang JX	Annals of Oncology (2014)	Abstract	Retrospective	Beijing, China	2008– 2009	Advanced HCC patients treated with cryoablation	150
Chen TM	Journal of Gastroenterology and Hepatology (2012)	Full-text	Retrospective	Taiwan, China	2003.7– 2010.12	Early HCC patients treated with RFA	158
Chen X	British Journal of Surgery (2012)	Full-text	Prospective	Hong Kong, China	2009.4– 2011.5	HCC patients with Child-Pugh grade A who underwent partial hepatectomy	190
Chen Z	Supportive Care in Cancer (2014)	Abstract	NA	Guangzhou, China	2008.9– 2010.6	Advanced HCC patients without fever or signs of infection	219
da Fonseca LG	Medical Oncology (2014)	Full-text	Retrospective	Sao Paulo, Brazil	2009.7– 2013.11	HCC patients who received sorafenib as initial systemic treatment	120
Dan J	PLoS ONE (2013)	Full-text	Retrospective	Guangzhou, China	2005.5– 2008.8	Small HCC patients treated with RFA	178
Facciorusso A	Journal of Gastroenterology and Hepatology (2014)	Full-text	NA	Foggia, Italy	2005.4– 2010.2	HCC patients treated with RFA	103
Fan W	PLoS ONE (2015)	Full-text	Retrospective	Guangzhou, China	2003.1– 2012.12	Recurrent HCC patients treated with TACE	132
Fu SJ	Medical Oncology (2013)	Full-text	NA	Guangzhou, China	2006.1– 2009.4	HBV-associated HCC patients treated with radical hepatectomy	282
Fu YP	Liver Cancer (2015)	Abstract	NA	Guangzhou, China	NA	HCC patients treated with curative resection	772
Gao F	Medicine (2015)	Full-text	Retrospective	Beijing, China	2008.10– 2012.5	Newly diagnosed with HCC	825
Gomez D; Farid S	World Journal of Surgery (2008); HPB (2010, Abstract)	Full-text	NA	Leeds, UK	1994.1– 2007.4	HCC patients treated with curative resection	96
Guo ZX	Chinese Journal of Cancer (2009)	Full-text	Retrospective	Guangzhou, China	2000– 2005	HCC patients treated with curative resection (age <35 years old)	91
Halazun KJ	Annals of Surgery (2009)	Full-text	Retrospective	NY, US	2001– 2007	HCC patients treated with LT	150
Harimoto N	Transplantation (2013)	Full-text	Retrospective	Fukuoka, Japan	1996.10– 2012.8	HCC patients treated with LDLT	167
Higashi T	Annals of Surgical Oncology (2015)	Full-text	Prospective	Kumamoto, Japan	2008– 2012	HCC patients treated with resection	215
Hu B	Clinical Cancer Research (2014)	Full-text	Retrospective/Prospective	Shanghai, China	2005– 2006/2010– 2011	HCC patients treated with curative resection	133/123
Huang GQ	Oncotarget (2015)	Full-text	Retrospective	Wenzhou, China	2007.1– 2014.1	HCC patients treated with curative resection	508
Huang J	Medical Oncology (2014)	Full-text	Prospective	Guangzhou, China	2008– 2009	HCC patients treated with hepatectomy as initial treatment	349
Huang ZL	Journal of Vascular and Interventional Radiology (2011)	Full-text	Retrospective	Guangzhou, China	2001– 2004	HCC patients treated with TACE	145
Kanno Y	Clinical Nutrition (2014)	Abstract	NA	Mibu, Japan	2000– 2012	HCC patients treated with curative surgery	418
Kim DG	Hepatology (2013)	Abstract	NA	Seoul, South Korea	2000.10– 2011.11	HCC patients treated with LDLT	224
Kinoshita A	Annals of Surgical Oncology (2015)	Full-text	Prospective; Retrospective	Tokyo, Japan	2005.1– 2012.8	Newly diagnosed HCC	186
Lai Q	Transplantation International (2014)	Full-text	NA	Brussels, Belgium	1994.1– 2012.3	Patients with pre-LT proven diagnosis of HCC who entered the waiting list for LT	181
Li C	Journal of Surgical Research (2015)	Full-text	NA	Chengdu, China	2007– 2014	HBV-associated HCC patients treated with resection	236
Li JP	Chinese Journal of Cancer Prevention and Treatment (2013)	Full-text	Retrospective	Jinan, China	2006.2– 2009.2	Unresectable HCC patients treated with TACE	154
Li X	Tumor Biology (2014)	Full-text	Retrospective	Guangzhou, China	2008.11– 2010.4	Advanced HCC patients (BCLC stages C and D) who did not receive sorafenib	205
Li X	PLoS ONE (2014)	Full-text	Retrospective	Beijing, China	2006.4– 2014.4	Recurrent HCC patients treated with curative thermal ablation	506
Liao R	World Journal of Surgical Oncology (2015)	Full-text	Retrospective	Chongqing, China	2007.1– 2010.12	Single-nodule small HCC patients treated with curative resection	222
Liao W	Translational Oncology (2014)	Full-text	Retrospective	Guilin, China	1999.9– 2007.6	HCC patients treated with curative resection	256
Liese J	Transplantation (2014)	Abstract	Retrospective	Frankfurt, Germany	2007.1– 2012.12	HCC patients treated with LT	92
Limaye AR	Hepatology Research (2013)	Full-text	Retrospective	FL, US	2000– 2008	HCC patients treated with LT	160
Long J	Hepatology International (2016)	Full-text	Prospective	Beijing, China	2010.8– 2014.7	HCC with PVTT patients treated with microwave ablation after TACE	60
Lu D	Transplantation (2015)	Abstract	NA	Hangzhou, China	2002– 2012	Small HCC patients treated with LT	140
Luè A	Journal of Hepatology (2014)	Abstract	NA	4 different hospitals, Spain	2005.8– 2013.10	HCC patients treated with sorafenib	186
Mano Y	Annals of Surgery (2013)	Full-text	Retrospective	3 different hospitals, Japan	1996.1– 20009.12	HCC patients treated with curative resection	958
McNally ME	Annals of Surgical Oncology (2013)	Full-text	Retrospective	OH, US	A 10–year period	HCC patients treated with TACE	104
Mizukoshi E	Hepatology (2015)	Abstract	NA	Kanazawa, Japan	NA	HCC patients treated with hepatic arterial infusion chemotherapy	36
Motomura T	Journal of Hepatology (2013)	Abstract	NA	Fukuoka, Japan	1999.7– 2011.3	HCC patients treated with LT	158
Na GH	World Journal of Gastroenterology (2014)	Full-text	Retrospective	Seoul, South Korea	2000.10– 2011.11	HCC patients treated with LDLT	224
Nagai S	Transplantation (2015)	Abstract	NA	IN, US	2001– 2012	HCC patients treated with LT	268
Ni XC	Medicine (2015)	Full-text	Retrospective	Shanghai, China	2010.12– 2012.1	HCC patients treated with resection (test cohort)	367
Oh BS	BMC Cancer (2013)	Full-text	Retrospective	Seoul, South Korea	2007.1– 2010.12	Newly diagnosed HCC	318
Okamura Y	World Journal of Surgery (2015)	Full-text	Retrospective	Shizuoka, Japan	2002.9– 2012.11	HCC patients treated with resection	256
Parisi I	Liver Transplantation (2014)	Full-text	NA	London, UK	1996– 2010	HCC patients treated with LT	150
Peng W	Journal of Surgical Research (2014)	Full-text	Retrospective	Chengdu, China	2007.2– 2012.3	Small HCC patients treated with curative resection	189
Pinato DJ	Translational Research (2012)	Full-text	Retrospective	London, UK	NA	HCC patients treated with TACE	54
Pinato DJ	Journal of Hepatology (2012)	Full-text	Retrospective	London, UK	1993– 2011	HCC patients (training set)	112
Ruan DY	World Journal of Gastroenterology (2015)	Full-text	Retrospective	Guangzhou, China	2003.9– 2011.6	HCC patients treated with curative resection	200
Shindoh J	Transplant International (2014)	Full-text	Retrospective	Tokyo, Japan	1996.1– 2012.12	HCC patients treated with LDLT	124
Sirin G	Hepatology International (2015)	Abstract	Retrospective	Kocaeli, Japan	2007– mid–2012	HCC patients treated with segmental resection and/or RFA	49
Sukato DC	Journal of Vascular and Interventional Radiology (2015)	Full-text	Retrospective	PA, US	2000.8– 2012.11	Intermediate- or advanced-stage HCC patients treated with radioembolization	176
Sullivan KM	Journal of Surgical Oncology (2014)	Full-text	NA	WI, US	2011.7– 2012.4	HCC patients	75
Sun Q	Biomedical Research (2014)	Full-text	Retrospective	Beijing, China	2003– 2008	HCC patients treated with resection	80
Tajiri K	Journal of Gastroenterology and Hepatology (2016)	Full-text	Retrospective	Toyama, Japan	2003– 2014	HCC patients treated with RFA	163
Tajiri K	Hepatology Research (2015)	Full-text	Retrospective	Toyama, Japan	2010– 2013	Advanced HCC patients treated with hepatic arterial infusion chemotherapy	26
Terashima T	Hepatology Research (2015)	Full-text	Retrospective	Ishikawa, Japan	2003.3– 2012.12	Advanced HCC patients treated with hepatic arterial infusion chemotherapy	266
Uchida K	American Journal of Transplantation (2012)	Abstract	NA	FL, US	2002.3– 2010.12	HCC patients treated with DDLT	275
Wang GY	PLoS ONE (2011)	Full-text	Retrospective	Guangzhou, China	2003.10– 2009.6	HBV-associated HCC patients treated with LT	101
Wang K	Liver Transplantation (2013)	Abstract	Retrospective	Hangzhou, China	NA	HCC patients treated with LT	235
Wang Q	Annals of Surgical Oncology (2015)	Full-text	NA	NY, US	1983– 2013	HBV-associated HCC patients treated with resection	234
Wang W	Hepatology Research (2015)	Full-text	Retrospective	Hangzhou, China	2002.1– 2012.12	Male HCC patients treated with LT	248
Wei K	Medical Oncology (2014)	Full-text	Retrospective	Tianjin, China	2010.1.1– 2013.5.31	Intermediate-advanced HCC patients treated with concurrent TAE in combination with sorafenib	40
Weinmann AJ	Hepatology (2015)	Abstract	Retrospective	Mainz, Germany	2007– 2013	HCC patients treated with sorafenib	148
Xiao GQ	Hepatobiliary and Pancreatic Diseases International (2015);	Full-text	Retrospective	Chengdu, China	1999.2– 2012.9	HCC patients treated with LT	305
Xu X	Chinese Medical Journal (2014)	Full-text	Retrospective	Xi'an, China	2003.7– 2012.9	HCC patients treated with TACE	178
Xue TC	Tumor Biology (2015)	Full-text	Retrospective	Shanghai, China	2008.1– 2011.3	Huge HCC patients treated with TACE	165
Yamamura K	Journal of Hepato-Biliary-Pancreatic Sciences (2014)	Full-text	Prospective	Aichi, Japan	2003.1– 2012.12	HCC patients treated with resection	113
Yang X	Chinese Journal of Radiology (2015)	Full-text	Retrospective	Chengdu, China	2000– 2010	HBV-associated HCC patients treated with TACE	546
Yang Z	Oncotarget (2015)	Full-text	Retrospective	Shanghai, China	2009.9– 2015.5	HBV-associated HCC patients treated with TACE	189
Yip V	HPB (2011)	Abstract	NA	Liverpool, UK	1997– 2008	HCC patients treated with resection	47
Yoshizumi T	Anticancer Research (2016)	Full-text	NA	Fukuoka, Japan	1999.4– 2015.3	HCC patients within Milan criteria treated with LDLT	129
Yoshizumi T	Transplantation Proceedings (2013)	Full-text	NA	Fukuoka, Japan	1999.4– 2011.12	HCC patients within Kyushu University criteria treated with LDLT	152
Yoshizumi T	Hepatology Research (2013)	Full-text	NA	Fukuoka, Japan	1999.4– 2012.8	Recurrent HCC adult patients treated with LDLT	104
Young AL	Journal of American College of Surgeons (2012)	Full-text	Retrospective	Leeds, UK	1994.1.1– 2008.12.31	HCC patients treated with resecction	142
Zhang J	Oncology Letters (2014)	Full-text	Retrospective	Wuhan, China	2002.3– 2012.8	Non-viral HCC patients treated with TACE	138
Zhang W	Medical Oncology (2015)	Full-text	Retrospective	Tianjin, China	2009.8.1– 2012.3.28	HCC patients who received sorafenib after resection	38
Zheng YB	Asian Pacific Journal of Cancer Prevention (2013)	Full-text	Retrospective	Guangzhou, China	2011.1– 2012.12	HCC patients treated with sorafenib monotherapy	65
Zheng YB	Chinese Journal of Interventional Imaging and Therapy (2013)	Full-text	Retrospective	Guangzhou, China	2008.1– 2012.12	HCC patients treated with TACE	77
Zhou D	Scientific Reports (2015)	Full-text	Retrospective	Guangzhou, China	2007– 2009	HCC patients treated with surgical resection, ablative therapy, and TACE	1061
Zhou DS	World Journal of Gastroenterology (2015)	Full-text	Retrospective	Guangzhou, China	2009.9– 2011.11	HBV–related HCC patients treated with TACE	224

Abbreviations: DDLT, deceased donor liver transplantation; HBV, hepatitis B virus; HCC, hepatocellular carcinoma; LDLT, living donor liver transplantation; LT, liver transplantation; RFA, radiofrequency ablation; TACE, transarterial chemoembolization.

Notes:

Some data from Kinoshita A, Annals of Surgical Oncology (2015) is also published by the same authors in British Journal of Cancer (2012).

Some data from Wang GY, PLoS ONE (2011) is also published by the same authors in National Medical Journal of China (2011).

Some data from Xiao GQ, Hepatobiliary and Pancreatic Diseases International (2015) is also published by the same authors in World Journal of Gastroenterology (2013) and Hepato-gastroenterology (2014).

### Study quality

Quality of included studies was summarized in Supplementary Table 1. Three, 18, 12, 30, and 27 studies had 7, 6, 5, 4, and ≤ 3 points, respectively.

### Meta analyses

### Association of baseline NLR with overall survival

**Figure 2:**
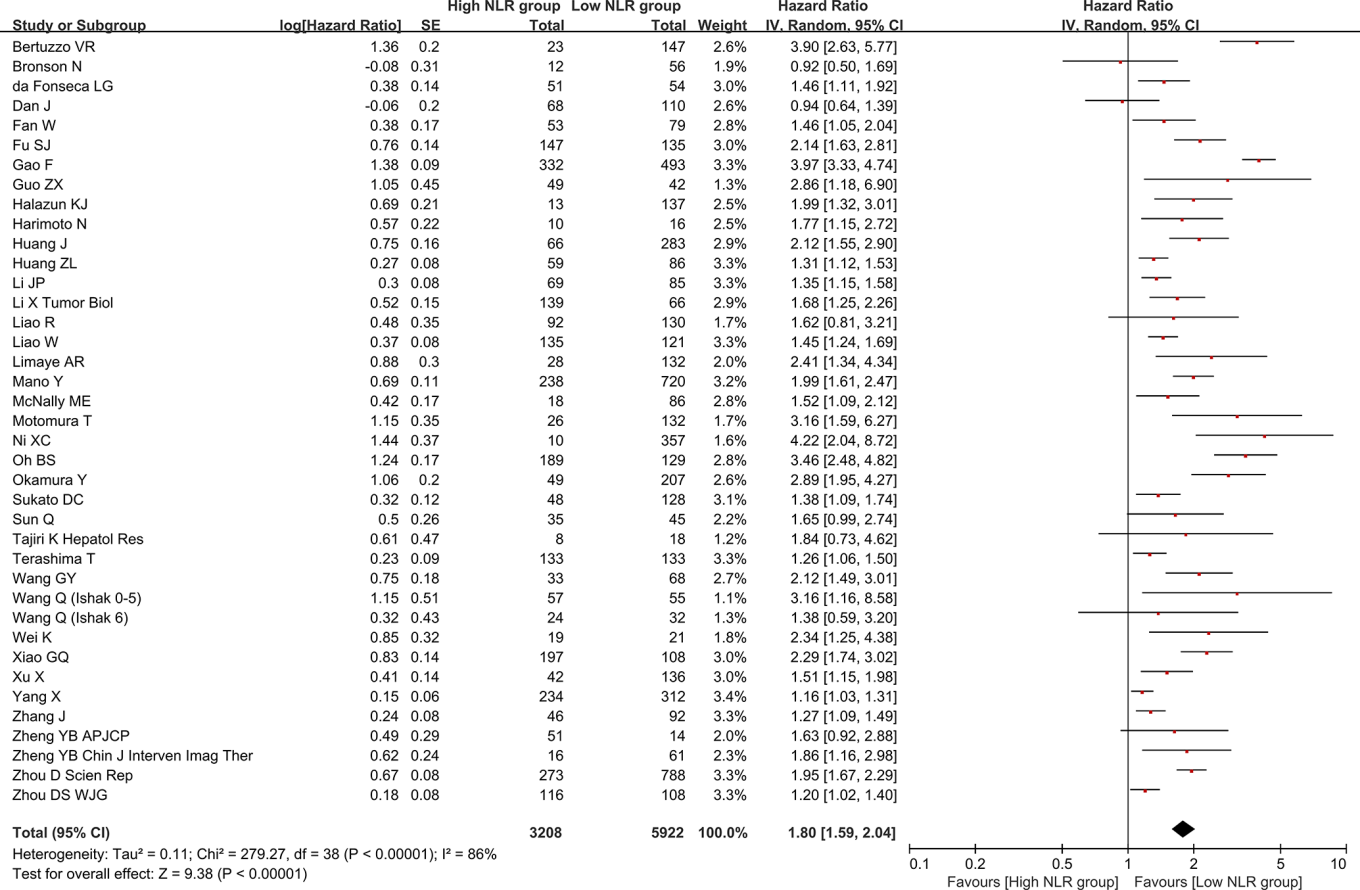
Forest plot evaluating the association between baseline NLR and overall survival in HCC patients

There were 39 groups of individual data regarding the association of baseline NLR with overall survival. They were extracted from 38 papers. HR was 1.80 (95% CI: 1.59–2.04, *p* < 0.00001), suggesting that low baseline NLR group had a significantly better overall survival than high baseline NLR group (Figure 2). Heterogeneity among studies was statistically significant (*I*^2^ = 86%, *p* < 0.00001). Funnel plot suggested a potential publication bias (Supplementary Figure 1).

### Association of post-treatment NLR with overall survival

**Figure 3:**
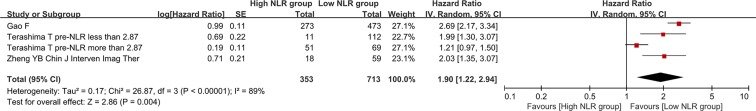
Forest plot evaluating the association between post-treatment NLR and overall survival in HCC patients

There were 4 groups of individual data regarding the association of post-treatment NLR with overall survival. They were extracted from 3 papers. HR was 1.90 (95% CI: 1.22–2.94, *p* = 0.004), suggesting that low post-treatment NLR group had a significantly better overall survival than high post-treatment NLR group (Figure 3). Heterogeneity among studies was statistically significant (*I*^2^ = 89%, *p* < 0.00001).

### Association of NLR change with overall survival

**Figure 4:**
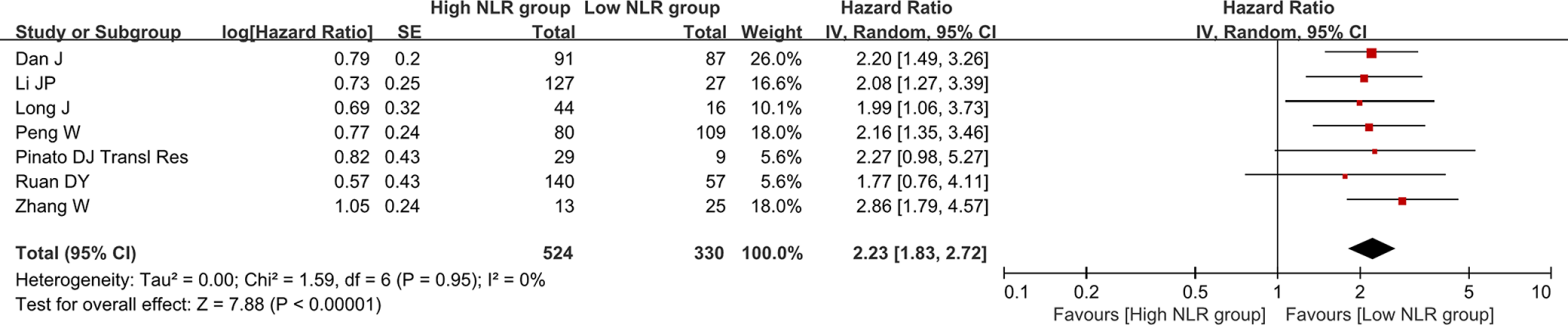
Forest plot evaluating the association between NLR change and overall survival in HCC patients

There were 7 groups of individual data regarding the association of NLR change with overall survival. They were extracted from 7 papers. HR was 2.23 (95% CI: 1.83–2.72, *p* < 0.00001), suggesting that decreased NLR group had a significantly better overall survival than increased NLR group (Figure 4). Heterogeneity among studies was not statistically significant (*I*^2^ = 0%, *p* = 0.95).

### Association of baseline NLR with recurrence-free or disease-free survival

**Figure 5:**
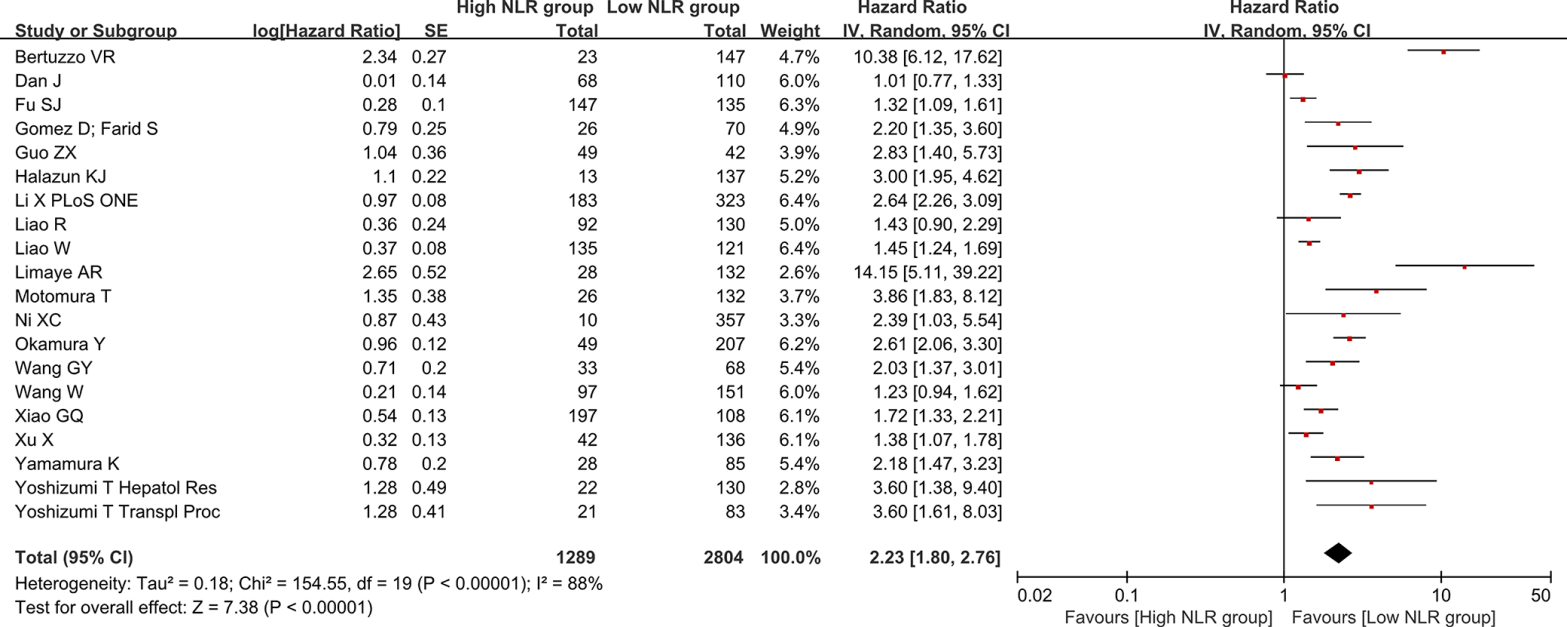
Forest plot evaluating the association between baseline NLR and recurrence-free or disease-free survival in HCC patients

There were 20 groups of individual data regarding the association of baseline NLR with recurrence-free or disease-free survival. They were extracted from 20 papers. HR was 2.23 (95% CI: 1.80–2.76, *p* < 0.00001), suggesting that low baseline NLR group had a significantly better recurrence-free or disease-free survival than high baseline NLR group (Figure 5). Heterogeneity among studies was statistically significant (*I*^2^ = 88%, *p* < 0.00001). Funnel plot suggested a potential publication bias (Supplementary Figure 2).

### Association of NLR change with recurrence-free or disease-free survival

**Figure 6:**
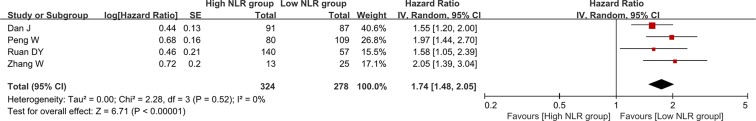
Forest plot evaluating the association between NLR change and recurrence-free or disease-free survival in HCC patients

There were 4 groups of individual data regarding the association of NLR change with recurrence-free or disease-free survival. They were extracted from 4 papers. HR was 2.23 (95% CI: 1.83–2.72, *p* < 0.00001), suggesting that decreased NLR group had a significantly better overall survival than increased NLR group (Figure 6). Heterogeneity among studies was not statistically significant (*I*^2^ = 0%, *p* = 0.52).

### Subgroup meta-analyses

Results of subgroup meta-analyses were summarized in Table 2.

**Table 2:** Results of subgroup meta-analyses

Items	No. groups of data	No. Pts in High NLR group	No. Pts in Low NLR group	Hazard ratio (95% CI)	*P* value	Heterogeneity	
						*I*^2^	*P* value
**Overall survival & baseline NLR**							
***Subgroup analysis according to treatment option***							
Liver transplantation	7	330	740	2.38 (1.95–2.91)	< 0.00001	38%	0.14
Surgical resection	12	914	2183	1.95 (1.61–2.37)	< 0.00001	62%	0.002
Radiofrequency ablation	1	68	110	0.94 (0.64–1.39)	0.76	NA	NA
Transarterial chemoembolization	9	653	1045	1.29 (1.20–1.38)	< 0.00001	14%	0.32
Radioembolization	1	48	128	1.38 (1.09–1.74)	0.008	NA	NA
Hepatic arterial infusion chemotherapy	2	141	151	1.28 (1.07–1.52)	0.006	0%	0.43
Transarterial embolization + sorafenib	1	19	21	2.34 (1.25–4.38)	0.008	NA	NA
Sorafenib	2	102	68	1.49 (1.17–1.91)	0.001	0%	0.73
Mixed	4	933	1476	2.59 (1.68–4.00)	< 0.0001	94%	< 0.00001
***Subgroup analysis according to NLR cut-off value range***							
NLR cut-off value ≥ 1, < 2	2	110	246	1.22 (0.77–1.93)	0.4	73%	0.05
NLR cut-off value ≥ 2, < 3	16	2077	3289	1.93 (1.56–2.39)	< 0.00001	91%	< 0.00001
NLR cut-off value ≥ 3, < 4	7	515	903	1.55 (1.28–1.88)	< 0.00001	75%	0.0005
NLR cut-off value = 4	6	308	349	2.07 (1.73–2.49)	< 0.00001	0%	0.63
NLR cut-off value = 5	8	198	1135	1.86 (1.37–2.52)	< 0.0001	83%	< 0.00001
***Subgroup analysis according to regions***							
America	8	251	680	1.55 (1.30–1.84)	< 0.00001	26%	0.22
Asia	30	2934	5095	1.81 (1.57–2.08)	< 0.00001	88%	< 0.00001
Europe	1	23	147	3.9 (2.63–5.77)	< 0.00001	NA	NA
**Overall survival & post-treatment NLR**							
***Subgroup analysis according to treatment option***							
Transarterial chemoembolization	1	18	59	2.03 (1.35–3.07)	0.0007	NA	NA
Hepatic arterial infusion chemotherapy	2	62	181	1.5 (0.92–2.43)	0.1	76%	0.04
Mixed	1	273	473	2.69 (2.17–3.34)	< 0.00001	NA	NA
***Subgroup analysis according to NLR cut-off value range***							
NLR cut-off value ≥ 2, < 3	3	335	654	1.86 (1.06–3.26)	0.03	92%	< 0.00001
NLR cut-off value = 4	1	18	59	2.03 (1.35–3.07)	0.0007	NA	NA
***Subgroup analysis according to regions***							
Asia	4	353	713	1.9 (1.22–2.94)	0.004	89%	< 0.00001
**Overall survival & NLR change**							
***Subgroup analysis according to treatment option***							
Surgical resection	2	220	166	2.06 (1.37–3.11)	0.0006	0%	0.68
Radiofrequency ablation	1	91	87	2.2 (1.49–3.26)	< 0.00001	NA	NA
Microwave ablation	1	44	16	1.99 (1.06–3.73)	0.03	NA	NA
Transarterial chemoembolization	2	156	36	2.12 (1.39–3.24)	0.0005	0%	0.86
Sorafenib	1	13	25	2.86 (1.79–4.57)	< 0.00001	NA	NA
***Subgroup analysis according to NLR cut-off value change***							
Increase or decrease	6	384	273	2.26 (1.84–2.78)	< 0.00001	0%	0.94
Delta	1	140	57	1.77 (0.76–4.11)	0.18	NA	NA
***Subgroup analysis according to regions***							
Asia	6	495	321	2.23 (1.81–2.74)	< 0.00001	0%	0.9
Europe	1	29	9	2.27 (0.98–5.27)	0.06	NA	NA
**RFS/DFS & baseline NLR**							
***Subgroup analysis according to treatment option***							
Liver transplantation	9	460	1088	3.31 (2.05–5.32)	< 0.00001	89%	< 0.00001
Surgical resection	8	536	1147	1.87 (1.47–2.37)	< 0.00001	76%	0.0002
Radiofrequency ablation	1	68	110	1.01 (0.77–1.33)	0.94	NA	NA
Thermal ablation	1	183	323	2.64 (2.26–3.09)	< 0.00001	NA	NA
Transarterial chemoembolization	1	42	136	1.38 (1.07–1.78)	0.01	NA	NA
***Subgroup analysis according to NLR cut-off value range***							
NLR cut-off value ≥ 1, < 2	2	110	246	1.18 (0.87–1.6)	0.27	62%	0.1
NLR cut-off value ≥ 2, < 3	6	655	958	1.9 (1.4–2.59)	< 0.0001	90%	< 0.00001
NLR cut-off value ≥ 3, < 4	3	158	304	1.72 (1.17–2.54)	0.006	73%	0.03
NLR cut-off value = 4	4	266	453	2.75 (1.63–4.63)	0.001	62%	0.05
NLR cut-off value = 5	5	100	843	4.51 (2.24–9.12)	< 0.0001	85%	< 0.0001
***Subgroup analysis according to regions***							
America	2	41	269	6.07 (1.34–27.55)	0.02	87%	0.006
Asia	16	1199	2318	1.85 (1.53–2.24)	< 0.00001	83%	< 0.00001
Europe	2	49	217	4.77 (1.04–21.77)	0.04	94%	< 0.0001
**RFS/DFS & NLR change**							
***Subgroup analysis according to treatment option***							
Surgical resection	2	220	166	1.82 (1.42–2.34)	< 0.00001	0%	0.4
Radiofrequency ablation	1	91	87	1.55 (1.20–2.00)	0.007	NA	NA
Sorafenib	1	13	25	2.05 (1.39–3.04)	0.0003	NA	NA
***Subgroup analysis according to NLR cut-off value change***							
Increase or decrease	3	184	221	1.77 (1.48–2.12)	< 0.00001	2%	0.36
Delta	1	140	57	1.58 (1.05–2.39)	0.03	NA	NA
***Subgroup analysis according to regions***							
Asia	4	324	278	1.74 (1.48–2.05)	< 0.00001	0%	0.52

## DISCUSSION

The present study systematically reviewed the role of NLR in the assessment of prognosis of HCC patients. To our knowledge, two previous meta-analyses also explored the association of NLR with prognosis of HCC [102–103]. Both of them were published in 2014. In the first meta-analysis, Xiao et al. searched the relevant literatures in August 2013 and identified 15 studies with 3,094 patients [102]. In the second meta-analysis, Xue et al. searched the relevant literatures in October 2013 and identified 26 studies with 4,461 patients [103]. Several advantages and features of our work should be acknowledged: 1) the relevant literatures were identified more recently (January 2016), and a larger number of relevant studies were included (90 papers with 20,475 patients); 2) according to the different time points when NLR values were obtained, we divided into baseline NLR, post-treatment NLR, and NLR change; 3) overall survival and recurrence-free or disease-free survival were selected as the primary outcomes; and 4) according to the treatment options, NLR cut-off values, and regions, we performed subgroup meta-analyses.

The major finding of our study was that low baseline NLR was significantly associated with better overall survival and recurrence-free or disease-free survival of HCC patients. This was based on a relatively large number of relevant data (38 papers for overall survival and 20 papers for recurrence-free or disease-free survival). Therefore, in our opinion, the relationship of baseline NLR with survival of HCC patients should be stable. This consideration was also confirmed by the subgroup meta-analyses: 1) except for one subgroup meta-analysis in patients undergoing radiofrequency ablation, other subgroup meta-analyses in patients undergoing different treatment modalities supported such an inverse association between them; 2) except for one subgroup meta-analysis with a NLR cut-off value of ≥ 1 and < 2, other subgroup meta-analyses with other NLR cut-off value ranges supported such an inverse association between them; and 3) regardless of regions, subgroup meta-analyses supported such an inverse association between them. Certainly, two following issues should be acknowledged. First, only one study focused on the patients undergoing radiofrequency ablation. Thus, more data might be necessary for the validation of our findings. Second, only two studies employed a NLR cut-off value of ≥ 1 and < 2. Given such a small NLR cut-off value, the survival difference between high and low NLR groups might be hardly achieved.

Another finding was that low post-treatment NLR was significantly associated with better overall survival of HCC patients. However, due to a small number of included studies, the subgroup meta-analyses were performed in patients undergoing transarterial chemoembolization and hepatic arterial infusion chemotherapy, studies with a NLR cut-off value of ≥ 2 and < 3 and NLR cut-off value of 4, and Asian studies. Except for one subgroup meta-analysis in HCC patients undergoing hepatic arterial infusion chemotherapy, other subgroup meta-analyses supported statistically significant associations. Similarly, we also found that decreased NLR after treatment was significantly associated with better recurrence-free or disease-free survival of HCC patients. Notably, such an inverse association was maintained regardless of treatment modalities.

Several limitations should be clarified. First, HR value for the association of NLR with overall survival was relatively small. Thus, their relationship might be weak. Whether the prognostic assessment of HCC can be guided by baseline NLR value should be further explored. Second, all included studies were observational, and most of them were retrospective. The quality of included studies was relatively low according to the NEWCASTLE-OTTAWA quality assessment scale. A major concern was a low comparability of patient characteristics between low and high NLR groups. This was primarily because all included studies were observational and NLR was only one of many variables included in univariate or multivariate analyses in a majority of original studies. Third, the heterogeneity was statistically significant in several meta-analyses. Random-effect model was employed to produce more conservative results. Fourth, because the researchers paid close attention on the prognostic role of NLR, some relevant paper has been published after this paper was finished [104].

In conclusion, the importance of NLR for assessing the overall survival and recurrence-free or disease-free survival should be acknowledged. Thus, we would like to suggest that NLR may be incorporated into the algorithm regarding the prognostic assessment of HCC. Further studies should confirm the prognostic ability of NLR in different specific settings according to the stage of HCC and treatment options and explore the superiority of NLR over other traditional prognostic scores or models. Additionally, considering that NLR change was associated with prognosis of HCC patients, future studies should explore how to prolong the survival of HCC patients by improving the inflammatory conditions.

## MATERIALS AND METHODS

We searched 3 major databases, including PubMed, EMBASE, and Cochrane library databases from the inception of databases. Search items were as follows: ((hepatocellular carcinoma) OR (liver cancer)) AND ((NLR) OR ((neutrophil) AND lymphocyte)). The last search was performed on January 20, 2016. All relevant literatures regarding the prognostic role of NLR in HCC patients were identified. Exclusion criteria were as follows: 1) duplicates; 2) comments; 3) erratum; 4) reviews; 5) case reports; 6) experimental studies; and 7) original studies did not evaluate the prognostic role of NLR in HCC patients. Publication language was not restricted.

We extracted the following data from the included studies: first author, journal, publication year, publication type, study design, regions, enrollment period, study population, number of patients, NLR cut-off values, and overall survival and recurrence-free or disease-free survival data according to the NLR value. In cases of uncertainty, we communicated with the authors and/or journal editors to validate the accuracy of data.

Given the nature of included studies, the study quality was assessed according to the NEWCASTLE-OTTAWA quality assessment scale for cohort studies [105]. This scale consisted of 8 questions with a maximum of 9 points. A study with more points would be of higher quality.

Data analysis was described as previously [106–108]. Briefly, only random-effects models were employed. Hazard ratios (HRs) were calculated because the overall survival and recurrence-free or disease-free survival were time-dependent data. I^2^ statistic and the Chi-square test were used to evaluate the heterogeneity among studies. Funnel plots were performed to evaluate the publication bias, if there were ≥ 10 groups of individual data included in the meta-analysis.

Notably, the meta-analyses were performed according to the times when NLR values were obtained (i.e. baseline NLR, post-treatment NLR, and NLR change). As for the baseline and post-treatment NLR, the patients were divided into two groups (i.e., low and high NLR group) according to the definitions of original studies. If the patients were divided into ≥ 3 groups in the original studies, the relevant data would not be included in the meta-analyses. Additionally, subgroup meta-analyses were performed according to the treatment options (i.e., liver transplantation, surgical resection, radiofrequency ablation, transarterial chemoembolization, radioembolization, hepatic arterial infusion chemotherapy, transarterial embolization plus sorafenib, sorafenib, and mixed treatments), NLR cut-off value ranges, and regions (i.e., America, Asia, and Europe).

## SUPPLEMENTARY MATERIALS FIGURES AND TABLE



## References

[1] Tandon P, Garcia-Tsao G (2009). Prognostic indicators in hepatocellular carcinoma: a systematic review of 72 studies. Liver Int.

[2] Forner A, Reig ME, de Lope CR, Bruix J (2010). Current strategy for staging and treatment: the BCLC update and future prospects. Semin Liver Dis.

[3] (1998). A new prognostic system for hepatocellular carcinoma: a retrospective study of 435 patients: the Cancer of the Liver Italian Program (CLIP) investigators. Hepatology.

[4] Yau T, Tang VY, Yao TJ, Fan ST, Lo CM, Poon RT (2014). Development of Hong Kong Liver Cancer staging system with treatment stratification for patients with hepatocellular carcinoma. Gastroenterology.

[5] Kudo M, Chung H, Osaki Y (2003). Prognostic staging system for hepatocellular carcinoma (CLIP score): its value and limitations, and a proposal for a new staging system, the Japan Integrated Staging Score (JIS score). J Gastroenterol.

[6] Child CG, Turcotte JG (1964). Surgery and portal hypertension. Major Probl Clin Surg.

[7] Johnson PJ, Berhane S, Kagebayashi C, Satomura S, Teng M, Reeves HL, O'Beirne J, Fox R, Skowronska A, Palmer D, Yeo W, Mo F, Lai P (2015). Assessment of liver function in patients with hepatocellular carcinoma: a new evidence-based approach-the ALBI grade. J Clin Oncol.

[8] Grivennikov SI, Greten FR, Karin M (2010). Immunity, inflammation, and cancer. Cell.

[9] Wu Y, Zhao Q, Peng C, Sun L, Li XF, Kuang DM (2011). Neutrophils promote motility of cancer cells via a hyaluronan-mediated TLR4/PI3K activation loop. J Pathol.

[10] Jablonska J, Leschner S, Westphal K, Lienenklaus S, Weiss S (2010). Neutrophils responsive to endogenous IFN-beta regulate tumor angiogenesis and growth in a mouse tumor model. J Clin Invest.

[11] Nind AP, Nairn RC, Rolland JM, Guli EP, Hughes ES (1973). Lymphocyte anergy in patients with carcinoma. Br J Cancer.

[12] Abdelmessih RM, Verna EC, Brubaker WD, Halazun KJ, Siegel A, Brown RS (2011). Hepatocellular carcinoma tumor staging at the time of liver transplant but not at diagnosis are predictive of tumor recurrence in patients who are downstaged with chemoembolization. Hepatology.

[13] Afshar M, Clarke H, Jackson-Wilding A, Ahmed A, Ma YT, Punia P (2015). Neutrophil lymphocyte ratio (NLR) at diagnosis is a predictor for survival in patients receiving sorafenib for advanced hepatocellular carcinoma (HCC): A large UK cohort. J Hepatol.

[14] Agopian VG, Harlander-Locke M, Zarrinpar A, Kaldas FM, Farmer DG, Yersiz H, Finn RS, Tong M, Hiatt JR, Busuttil RW (2015). A novel prognostic nomogram accurately predicts hepatocellular carcinoma recurrence after liver transplantation: Analysis of 865 consecutive liver transplant recipients. J Am Coll Surg.

[15] Aino H, Sumie S, Niizeki T, Kuromatsu R, Tajiri N, Nakano M, Satani M, Yamada S, Okamura S, Shimose S, Sumie H, Torimura T, Sata M (2014). Clinical characteristics and prognostic factors for advanced hepatocellular carcinoma with extrahepatic metastasis. Mol Clin Oncol.

[16] Bertuzzo VR, Cescon M, Ravaioli M, Grazi GL, Ercolani G, Del Gaudio M, Cucchetti A, D'Errico-Grigioni A, Golfieri R, Pinna AD (2011). Analysis of factors affecting recurrence of hepatocellular carcinoma after liver transplantation with a special focus on inflammation markers. Transplantation.

[17] Bodzin A, Reino D, Lunsford K, Harlander-Locke M, Markovic D, Zarrinpar A, Kaldas F, Farmer D, Yersiz H, Busuttil R, Agopian V (2015). Predictors of time to recurrence and mortality in patients developing recurrent hepatocellular carcinoma after liver transplantation. Am J Transplant.

[18] Bronson N, Enestvedt K, Thomas E, Vermette D, Orloff S (2012). Preoperative neutrophil to lymphocyte ratio does not predict recurrence or prognosis after resection for hepatocellular carcinoma. HPB.

[19] Bruixola G, Niño OM, Diaz-Beveridge R, Reche E, Salvador C, Escoin C, Akhoundova D, Segura A, Gimenez A, Aparicio J (2015). Baseline neutrophil-to-lymphocyte ratio (NLR) and early toxicity as prognostic factors in advanced hepatocellular carcinoma patients treated with sorafenib. J Clin Oncol.

[20] Chan AW, Chan SL, Wong GL, Wong VW, Chong CC, Lai PB, Chan HL, To KF (2015). Prognostic Nutritional Index (PNI) Predicts Tumor Recurrence of Very Early/Early Stage Hepatocellular Carcinoma After Surgical Resection. Ann Surg Oncol.

[21] Chang JX, Zeng Z, Lu YY, Qu HJ, Xu LG, Gao X, Wang H, Lou M, Wang C, Yang Y (2014). Nlr of pro-cryoablation can predict the prognosis of patients with advanced hepatocellular carcinoma. Ann Oncol.

[22] Chen TM, Lin CC, Huang PT, Wen CF (2012). Neutrophil-to-lymphocyte ratio associated with mortality in early hepatocellular carcinoma patients after radiofrequency ablation. J Gastroenterol Hepatol.

[23] Chen X, Zhai J, Cai X, Zhang Y, Wei L, Shi L, Wu D, Shen F, Lau WY, Wu M (2012). Severity of portal hypertension and prediction of postoperative liver failure after liver resection in patients with Child-Pugh grade A cirrhosis. Br J Surg.

[24] Chen Z, Xy LI, Min DONG, Qu L, Xiaokun M, Xiangyuan WU (2014). Identification of prognostic value of neutrophil-to-lymphocyte ratio (NLR) in patients with advanced hepatocellular carcinoma (HCC). Support Care Cancer.

[25] da Fonseca LG, Barroso-Sousa R, Bento Ada S, Blanco BP, Valente GL, Pfiffer TE, Hoff PM, Sabbaga J (2014). Pre-treatment neutrophil-to-lymphocyte ratio affects survival in patients with advanced hepatocellular carcinoma treated with sorafenib. Med Oncol.

[26] Dan J, Zhang Y, Peng Z, Huang J, Gao H, Xu L, Chen M (2013). Postoperative Neutrophil-to-Lymphocyte Ratio Change Predicts Survival of Patients with Small Hepatocellular Carcinoma Undergoing Radiofrequency Ablation. PLoS ONE.

[27] Facciorusso A, Del Prete V, Antonino M, Neve V, Crucinio N, Di Leo A, Carr BI, Barone M (2014). Serum ferritin as a new prognostic factor in hepatocellular carcinoma patients treated with radiofrequency ablation. J Gastroenterol Hepatol.

[28] Fan W, Zhang Y, Wang Y, Yao X, Yang J, Li J (2015). Neutrophil-to-lymphocyte and platelet-to-lymphocyte ratios as predictors of survival and metastasis for recurrent hepatocellular carcinoma after transarterial chemoembolization. PLoS ONE.

[29] Fu SJ, Shen SL, Li SQ, Hua YP, Hu WJ, Liang LJ, Peng BG (2013). Prognostic value of preoperative peripheral neutrophil-to-lymphocyte ratio in patients with HBV-associated hepatocellular carcinoma after radical hepatectomy. Med Oncol.

[30] Fu YP, Ni XC, Yi Y, Cai XY, He HW, Wang JX, Lu ZF, Zhou J, Fan J, Qiu SJ (2015). A novel and validated inflammation based score (IBS) derived from neutrophil to lymphocyte ratio predicts survival in patients with hepatocellular carcinoma following curative surgical resection. Liver Cancer.

[31] Gao F, Li X, Geng M, Ye X, Liu H, Liu Y, Wan G, Wang X (2015). Pretreatment neutrophil-lymphocyte ratio: an independent predictor of survival in patients with hepatocellular carcinoma. Medicine.

[32] Gomez D, Farid S, Malik HZ, Young AL, Toogood GJ, Lodge JP, Prasad KR (2008). Preoperative neutrophil-to-lymphocyte ratio as a prognostic predictor after curative resection for hepatocellular carcinoma. World J Surg.

[33] Guo ZX, Wei W, Zhong C, Shi M, Chen MS, Guo RP (2009). Correlation of preoperative neutrophil-to-lymphocyte ratio to prognosis of young patients with hepatocellular carcinoma. Chin J Cancer.

[34] Halazun KJ, Hardy MA, Rana AA, Woodland DCt, Luyten EJ, Mahadev S, Witkowski P, Siegel AB, Brown RS, Emond JC (2009). Negative impact of neutrophil-lymphocyte ratio on outcome after liver transplantation for hepatocellular carcinoma. Ann Surg.

[35] Harimoto N, Shirabe K, Nakagawara H, Toshima T, Yamashita Y, Ikegami T, Yoshizumi T, Soejima Y, Ikeda T, Maehara Y (2013). Prognostic factors affecting survival at recurrence of hepatocellular carcinoma after living-donor liver transplantation: with special reference to neutrophil/lymphocyte ratio. Transplantation.

[36] Higashi T, Hayashi H, Kaida T, Arima K, Takeyama H, Taki K, Izumi D, Tokunaga R, Kosumi K, Nakagawa S, Okabe H, Imai K, Nitta H (2015). Prognostic Impact of Visceral Fat Amount and Branched-Chain Amino Acids (BCAA) in Hepatocellular Carcinoma. Ann Surg Oncol.

[37] Hu B, Yang XR, Xu Y, Sun YF, Sun C, Guo W, Zhang X, Wang WM, Qiu SJ, Zhou J, Fan J (2014). Systemic immune-inflammation index predicts prognosis of patients after curative resection for hepatocellular carcinoma. Clin Cancer Res.

[38] Huang GQ, Zhu GQ, Liu YL, Wang LR, Braddock M, Zheng MH, Zhou MT (2016). Stratified neutrophil-to-lymphocyte ratio accurately predict mortality risk in hepatocellular carcinoma patients following curative liver resection. Oncotarget.

[39] Huang J, Xu L, Luo Y, He F, Zhang Y, Chen M (2014). The inflammation-based scores to predict prognosis of patients with hepatocellular carcinoma after hepatectomy. Med Oncol.

[40] Huang ZL, Luo J, Chen MS, Li JQ, Shi M (2011). Blood neutrophil-to-lymphocyte ratio predicts survival in patients with unresectable hepatocellular carcinoma undergoing transarterial chemoembolization. J Vasc Interv Radiol.

[41] Kanno Y, Ishizuka M, Kubota K (2014). Neutrophil to lymphocyte ratio is associated with postoperative survival of primary hepatocellular carcinoma patients undergoing curative surgery. Clin Nutr.

[42] Kim DG (2013). Neutrophil-lymphocyte ratio and serum C-reactive protein predict overall and recurrence free survival after liver transplantation in patients with hepatocellular carcinoma. Hepatology.

[43] Kinoshita A, Onoda H, Imai N, Iwaku A, Oishi M, Tanaka K, Fushiya N, Koike K, Nishino H, Matsushima M (2015). The C-Reactive Protein/Albumin Ratio, a Novel Inflammation-Based Prognostic Score, Predicts Outcomes in Patients with Hepatocellular Carcinoma. Ann Surg Oncol.

[44] Lai Q, Castro Santa E, Rico Juri JM, Pinheiro RS, Lerut J (2014). Neutrophil and platelet-to-lymphocyte ratio as new predictors of dropout and recurrence after liver transplantation for hepatocellular cancer. Transpl Int.

[45] Li C, Wen TF, Yan LN, Li B, Wang WT, Yang JY, Xu MQ (2015). Postoperative neutrophil-to-lymphocyte ratio plus platelet-to-lymphocyte ratio predicts the outcomes of hepatocellular carcinoma. J Surg Res.

[46] Li JP, Hu SL, Chen H, Bu WZ, Song JL (2013). Blood neutrophil-to-lymphocyte ratio predicts survival in patients with unresectable hepatocellular carcinoma undergoing transarterial chemoembolization. Chinese Journal of Cancer Prevention and Treatment.

[47] Li X, Chen ZH, Ma XK, Chen J, Wu DH, Lin Q, Dong M, Wei L, Wang TT, Ruan DY, Lin ZX, Xing YF, Deng Y (2014). Neutrophil-to-lymphocyte ratio acts as a prognostic factor for patients with advanced hepatocellular carcinoma. Tumour Biol.

[48] Li X, Han Z, Cheng Z, Yu J, Liu S, Yu X, Liang P (2014). Preoperative neutrophil-to-lymphocyte ratio is a predictor of recurrence following thermal ablation for recurrent hepatocellular carcinoma: A retrospective analysis. PLoS ONE.

[49] Liao R, Tang ZW, Li DW, Luo SQ, Huang P, Du CY (2015). Preoperative neutrophil-to-lymphocyte ratio predicts recurrence of patients with single-nodule small hepatocellular carcinoma following curative resection: A retrospective report. World J Surg Oncol.

[50] Liao W, Zhang J, Zhu Q, Qin L, Yao W, Lei B, Shi W, Yuan S, Tahir SA, Jin J, He S (2014). Preoperative neutrophil-to-lymphocyte ratio as a new prognostic marker in hepatocellular carcinoma after curative resection. Transl Oncol.

[51] Liese J, Peveling-Oberhag J, Moench C, Schnitzbauer A, Welker M, Zeuzem S, Bechstein W, Ulrich F (2014). A possible role of miRNAs as predicitve markers for the recurrence of hepatocellular carcinoma after liver transplantation. Transplantation.

[52] Limaye AR, Clark V, Soldevila-Pico C, Morelli G, Suman A, Firpi R, Nelson DR, Cabrera R (2013). Neutrophil-lymphocyte ratio predicts overall and recurrence-free survival after liver transplantation for hepatocellular carcinoma. Hepatol Res.

[53] Long J, Zheng JS, Sun B, Lu N (2016). Microwave ablation of hepatocellular carcinoma with portal vein tumor thrombosis after transarterial chemoembolization: a prospective study. Hepatol Int.

[54] Lu D, Xu X, Zheng S (2015). The prognostic capacity of micro-vascular invasion in liver transplantation for small hepatocellular carcinoma. Transplantation.

[55] Luè A, Bustamante FJ, Iñarrairaegui M, Arenas JI, Serrano MT, Testillano M, Lorente S, Gil C, De La Torre M, Gómez A, Sangro B (2014). Neutrophil-to-lymphocyte ratio is a predictor of one-year survival in patients with hepatocellular carcinoma receiving sorafenib. J Hepatol.

[56] Mano Y, Shirabe K, Yamashita Y, Harimoto N, Tsujita E, Takeishi K, Aishima S, Ikegami T, Yoshizumi T, Yamanaka T, Maehara Y (2013). Preoperative neutrophil-to-lymphocyte ratio is a predictor of survival after hepatectomy for hepatocellular carcinoma: a retrospective analysis. Ann Surg.

[57] McNally ME, Martinez A, Khabiri H, Guy G, Michaels AJ, Hanje J, Kirkpatrick R, Bloomston M, Schmidt CR (2013). Inflammatory markers are associated with outcome in patients with unresectable hepatocellular carcinoma undergoing transarterial chemoembolization. Ann Surg Oncol.

[58] Mizukoshi E, Yamashita T, Arai K, Terashima T, Kitahara M, Nakagawa H, Iida N, Fushimi K, Kaneko S (2015). Myeloid-derived suppressor cells correlate with patient outcomes in hepatic arterial infusion chemotherapy for hepatocellular carcinoma. Hepatology.

[59] Motomura T, Shirabe K, Mano Y, Muto J, Toshima T, Umemoto Y, Fukuhara T, Uchiyama H, Ikegami T, Yoshizumi T, Soejima Y, Maehara Y (2013). Neutrophil-lymphocyte ratio reflects hepatocellular carcinoma recurrence after liver transplantation via inflammatory microenvironment. J Hepatol.

[60] Na GH, Kim DG, Han JH, Kim EY, Lee SH, Hong TH, You YK (2014). Inflammatory markers as selection criteria of hepatocellular carcinoma in living-donor liver transplantation. World J Gastroenterol.

[61] Nagai S, Mangus RS, Frost E, Kubal CA, Ekser B, Fridell JA, Kwo P, Maluccio MA, Tector AJ (2015). Predictors for outcome of recurrence of hepatocellular carcinoma after liver transplantation: Successful treatment and long survival. Transplantation.

[62] Ni XC, Yi Y, Fu YP, He HW, Cai XY, Wang JX, Zhou J, Cheng YF, Jin JJ, Fan J, Qiu SJ (2015). Prognostic value of the modified glasgow prognostic score in patients undergoing radical surgery for hepatocellular carcinoma. Medicine.

[63] Oh BS, Jang JW, Kwon JH, You CR, Chung KW, Kay CS, Jung HS, Lee S (2013). Prognostic value of C-reactive protein and neutrophil-to-lymphocyte ratio in patients with hepatocellular carcinoma. BMC Cancer.

[64] Okamura Y, Ashida R, Ito T, Sugiura T, Mori K, Uesaka K (2015). Preoperative neutrophil to lymphocyte ratio and prognostic nutritional index predict overall survival after hepatectomy for hepatocellular carcinoma. World J Surg.

[65] Parisi I, Tsochatzis E, Wijewantha H, Rodriguez-Peralvarez M, De Luca L, Manousou P, Fatourou E, Pieri G, Papastergiou V, Davies N, Yu D, Luong T, Dhillon AP (2014). Inflammation-based scores do not predict post-transplant recurrence of hepatocellular carcinoma in patients within Milan criteria. Liver Transpl.

[66] Peng W, Li C, Wen TF, Yan LN, Li B, Wang WT, Yang JY, Xu MQ (2014). Neutrophil to lymphocyte ratio changes predict small hepatocellular carcinoma survival. J Surg Res.

[67] Pinato DJ, Sharma R (2012). An inflammation-based prognostic index predicts survival advantage after transarterial chemoembolization in hepatocellular carcinoma. Transl Res.

[68] Pinato DJ, Stebbing J, Ishizuka M, Khan SA, Wasan HS, North BV, Kubota K, Sharma R (2012). A novel and validated prognostic index in hepatocellular carcinoma: The inflammation based index (IBI). J Hepatol.

[69] Ruan DY, Lin ZX, Li Y, Jiang N, Li X, Wu DH, Wang TT, Chen J, Lin Q, Wu XY (2015). Poor oncologic outcomes of hepatocellular carcinoma patients with intra-abdominal infection after hepatectomy. World J Gastroenterol.

[70] Shindoh J, Sugawara Y, Nagata R, Kaneko J, Tamura S, Aoki T, Sakamoto Y, Hasegawa K, Tanaka T, Kokudo N (2014). Evaluation methods for pretransplant oncologic markers and their prognostic impacts in patient undergoing living donor liver transplantation for hepatocellular carcinoma. Transpl Int.

[71] Sirin G, Senturk O, Yilmaz H, Celebi A, Hulagu S (2015). Are inflammatory markers more useful than noninvasive fibrosis panels for prediction of hepatocellular carcinoma recurrence. Hepatol Int.

[72] Sukato DC, Tohme S, Chalhoub D, Han K, Zajko A, Amesur N, Orons P, Marsh JW, Geller DA, Tsung A (2015). The prognostic role of neutrophil-to-lymphocyte ratio in patients with unresectable hepatocellular carcinoma treated with radioembolization. J Vasc Interv Radiol.

[73] Sullivan KM, Groeschl RT, Turaga KK, Tsai S, Christians KK, White SB, Rilling WS, Pilgrim CH, Gamblin TC (2014). Neutrophil-to-lymphocyte ratio as a predictor of outcomes for patients with hepatocellular carcinoma: a Western perspective. J Surg Oncol.

[74] Sun Q, Jiao SC, Wu JY, Long YY, Chen L (2014). Pretreatment haematological laboratory values: The new prognostic factors in patients undergoing hepatectomy for hepatocellular carcinoma. Biomedical Research (India).

[75] Tajiri K, Baba H, Kawai K, Minemura M, Yasumura S, Takahara T, Sugiyama T (2016). Neutrophil-to-lymphocyte ratio predicts recurrence after radiofrequency ablation in hepatitis B virus infection. J Gastroenterol Hepatol.

[76] Tajiri K, Kawai K, Minemura M, Yasumura S, Hosokawa A, Kawabe H, Tomizawa G, Sugiyama T (2015). Neutrophil/lymphocyte ratio as a prognostic indicator of hepatic arterial infusion chemotherapy with arterial cisplatin plus continuous 5-fluorouracil. Hepatol Res.

[77] Terashima T, Yamashita T, Iida N, Nakagawa H, Arai K, Kitamura K, Kagaya T, Sakai Y, Mizukoshi E, Honda M, Kaneko S (2015). Blood neutrophil to lymphocyte ratio as a predictor in patients with advanced hepatocellular carcinoma treated with hepatic arterial infusion chemotherapy. Hepatol Res.

[78] Uchida K, Levi D, Nishida S, Selvaggi G, Tekin A, Taizo H, Garcia M, Feun L, Tzakis AG (2012). Recurrence pattern and prognosis after liver transplant for hepatocellular carcinoma in the MELD ERA. Am J Transplant.

[79] Wang GY, Yang Y, Li H, Zhang J, Jiang N, Li MR, Zhu HB, Zhang Q, Chen GH (2011). A scoring model based on neutrophil to lymphocyte ratio predicts recurrence of HBV-associated hepatocellular carcinoma after liver transplantation. PLoS ONE.

[80] Wang K, Ling Q, Xu X, Wei Q, Chen J, Wu L, Zheng S (2013). Does neutrophil to lymphocyte ratio improve hangzhou criteria for predicting tumor recurrence after liver transplantation?. Liver Transplant.

[81] Wang Q, Blank S, Fiel MI, Kadri H, Luan W, Warren L, Zhu A, Deaderick PA, Sarpel U, Labow DM, Hiotis SP (2015). The Severity of Liver Fibrosis Influences the Prognostic Value of Inflammation-Based Scores in Hepatitis B-Associated Hepatocellular Carcinoma. Ann Surg Oncol.

[82] Wang W, Ye Y, Wang T, Zhang F, Geng L, Yu J, Zhou L, Yan S, Zheng S (2015). Prognostic prediction of male recipients selected for liver transplantation: With special attention to neutrophil-lymphocyte ratio. Hepatol Res.

[83] Wei K, Wang M, Zhang W, Mu H, Song TQ (2014). Neutrophil-lymphocyte ratio as a predictor of outcomes for patients with hepatocellular carcinoma undergoing TAE combined with Sorafenib. Med Oncol.

[84] Weinmann AJ, Koch S, Sprinzl MF, Galle PR, Wörns M (2015). Prognostic significance of inflammation based scores for patients with hepatocellular carcinoma treated with sorafenib in a western collective. Hepatology.

[85] Xiao GQ, Yang JY, Yan LN (2015). Combined Hangzhou criteria with neutrophil-lymphocyte ratio is superior to other criteria in selecting liver transplantation candidates with HBV-related hepatocellular carcinoma. Hepatobiliary Pancreat Dis Int.

[86] Xu X, Chen W, Zhang L, Miao R, Zhou Y, Wan Y, Dong Y, Liu C (2014). Prognostic significance of neutrophil to lymphocyte ratio in patients with hepatocellular carcinoma after transcatheter arterial chemoembolization. Chin Med J (Engl).

[87] Xue TC, Jia QA, Ge NL, Chen Y, Zhang BH, Ye SL (2015). Imbalance in systemic inflammation and immune response following transarterial chemoembolization potentially increases metastatic risk in huge hepatocellular carcinoma. Tumour Biol.

[88] Yamamura K, Sugimoto H, Kanda M, Yamada S, Nomoto S, Nakayama G, Fujii T, Koike M, Fujiwara M, Kodera Y (2014). Comparison of inflammation-based prognostic scores as predictors of tumor recurrence in patients with hepatocellular carcinoma after curative resection. J Hepatobiliary Pancreat Sci.

[89] Yang X, Zhou S, Wen H, Li W, Li Z, Wu G, Sun Y, Wu H, Xu G (2015). Preoperative neutrophil-lymphocyte ratio as a prognostic predictor after transarterial chemoembolization for HBV-associated hepatocellular carcinoma. Chin J Radiol.

[90] Yang Z, Zhang J, Lu Y, Xu Q, Tang B, Wang Q, Zhang W, Chen S, Lu L, Chen X (2015). Aspartate aminotransferase-lymphocyte ratio index and systemic immune-inflammation index predict overall survival in HBV-related hepatocellular carcinoma patients after transcatheter arterial chemoembolizations. Oncotarget.

[91] Yip V, Fenwick S, Malik H, Ghaneh P, Sarno G, Wu A, Terlizzo M, Poston G (2011). Resection of hepatocellular carcinoma arising in noncirrhotic/non-fibrotic liver. HPB.

[92] Yoshizumi T, Harimoto N, Itoh S, Okabe H, Kimura K, Uchiyama H, Ikegami T, Ikeda T, Maehara Y (2016). Living Donor Liver Transplantation for Hepatocellular Carcinoma within Milan Criteria in the Present Era. Anticancer Res.

[93] Yoshizumi T, Ikegami T, Toshima T, Harimoto N, Uchiyama H, Soejima Y, Yamashita Y, Shirabe K, Maehara Y (2013). Two-step selection criteria for living donor liver transplantation in patients with hepatocellular carcinoma. Transplant Proc.

[94] Yoshizumi T, Ikegami T, Yoshiya S, Motomura T, Mano Y, Muto J, Ikeda T, Soejima Y, Shirabe K, Maehara Y (2013). Impact of tumor size, number of tumors and neutrophil-to-lymphocyte ratio in liver transplantation for recurrent hepatocellular carcinoma. Hepatol Res.

[95] Young AL, Adair R, Prasad KR, Toogood GJ, Lodge JP (2012). Hepatocellular carcinoma within a noncirrhotic, nonfibrotic, seronegative liver: surgical approaches and outcomes. J Am Coll Surg.

[96] Zhang J, Gong F, Li L, Zhao M, Song J (2014). Diabetes mellitus and the neutrophil to lymphocyte ratio predict overall survival in non-viral hepatocellular carcinoma treated with transarterial chemoembolization. Oncol Lett.

[97] Zhang W, Zhao G, Wei K, Zhang Q, Ma W, Wu Q, Zhang T, Kong D, Li Q, Song T (2015). Adjuvant sorafenib therapy in patients with resected hepatocellular carcinoma: evaluation of predictive factors. Med Oncol.

[98] Zheng YB, Zhao W, Liu B, Li Y, Hu BS, Lu LG (2013). Prognostic significance of blood neutrophil-to-lymphocyte ratio in patients with hepatocellular carcinoma undergoing TACE. Chinese Journal of Interventional Imaging and Therapy.

[99] Zheng YB, Zhao W, Liu B, Lu LG, He X, Huang JW, Li Y, Hu BS (2013). The blood neutrophil-to-lymphocyte ratio predicts survival in patients with advanced hepatocellular carcinoma receiving sorafenib. Asian Pac J Cancer Prev.

[100] Zhou D, Zhang Y, Xu L, Zhou Z, Huang J, Chen M (2015). A monocyte/granulocyte to lymphocyte ratio predicts survival in patients with hepatocellular carcinoma. Sci Rep.

[101] Zhou DS, Xu L, Luo YL, He FY, Huang JT, Zhang YJ, Chen MS (2015). Inflammation scores predict survival for hepatitis B virus-related hepatocellular carcinoma patients after transarterial chemoembolization. World J Gastroenterol.

[102] Xiao WK, Chen D, Li SQ, Fu SJ, Peng BG, Liang LJ (2014). Prognostic significance of neutrophil-lymphocyte ratio in hepatocellular carcinoma: A meta-analysis. BMC Cancer.

[103] Xue TC, Zhang L, Xie XY, Ge NL, Li LX, Zhang BH, Ye SL, Ren ZG (2014). Prognostic significance of the neutrophil-to-lymphocyte ratio in primary liver cancer: A meta-analysis. PLoS ONE.

[104] Lu SD, Wang YY, Peng NF, Peng YC, Zhong JH, Qin HG, Xiang BD, You XM, Ma L, Li LQ (2016). Preoperative Ratio of Neutrophils to Lymphocytes Predicts Postresection Survival in Selected Patients With Early or Intermediate Stage Hepatocellular Carcinoma. Medicine.

[105] Wells GA, Shea B, O'Connell D, Peterson J, Welch V, Losos M, Tugwell P The Newcastle-Ottawa Scale (NOS) for assessing the quality of nonrandomised studies in meta-analyses. http://wwwohrica/programs/clinical_epidemiology/oxfordasp.

[106] Qi X, Liu L, Wang D, Li H, Su C, Guo X (2015). Hepatic resection alone versus in combination with pre- and post-operative transarterial chemoembolization for the treatment of hepatocellular carcinoma: A systematic review and meta-analysis. Oncotarget.

[107] Qi X, Wang D, Su C, Li H, Guo X (2015). Hepatic resection versus transarterial chemoembolization for the initial treatment of hepatocellular carcinoma: A systematic review and meta-analysis. Oncotarget.

[108] Qi X, Tang Y, An D, Bai M, Shi X, Wang J, Han G, Fan D (2014). Radiofrequency ablation versus hepatic resection for small hepatocellular carcinoma: a meta-analysis of randomized controlled trials. J Clin Gastroenterol.

