# Supplementary material for: Neutrophil-to-lymphocyte ratio for the prognostic assessment of hepatocellular carcinoma: A systematic review and meta-analysis of observational studies
Source: Oncotarget. 2016 Jun 10;7(29):45283–301. doi: 10.18632/oncotarget.9942 (PMC5216723; doi:10.18632/oncotarget.9942)
Supplement: Supplementary file 1 [file oncotarget-07-45283-s001.pdf]

Neutrophil-to-lymphocyte ratio for the prognostic assessment of hepatocellular carcinoma: A systematic review and meta-analysis of observational studies

Supplementary Materials

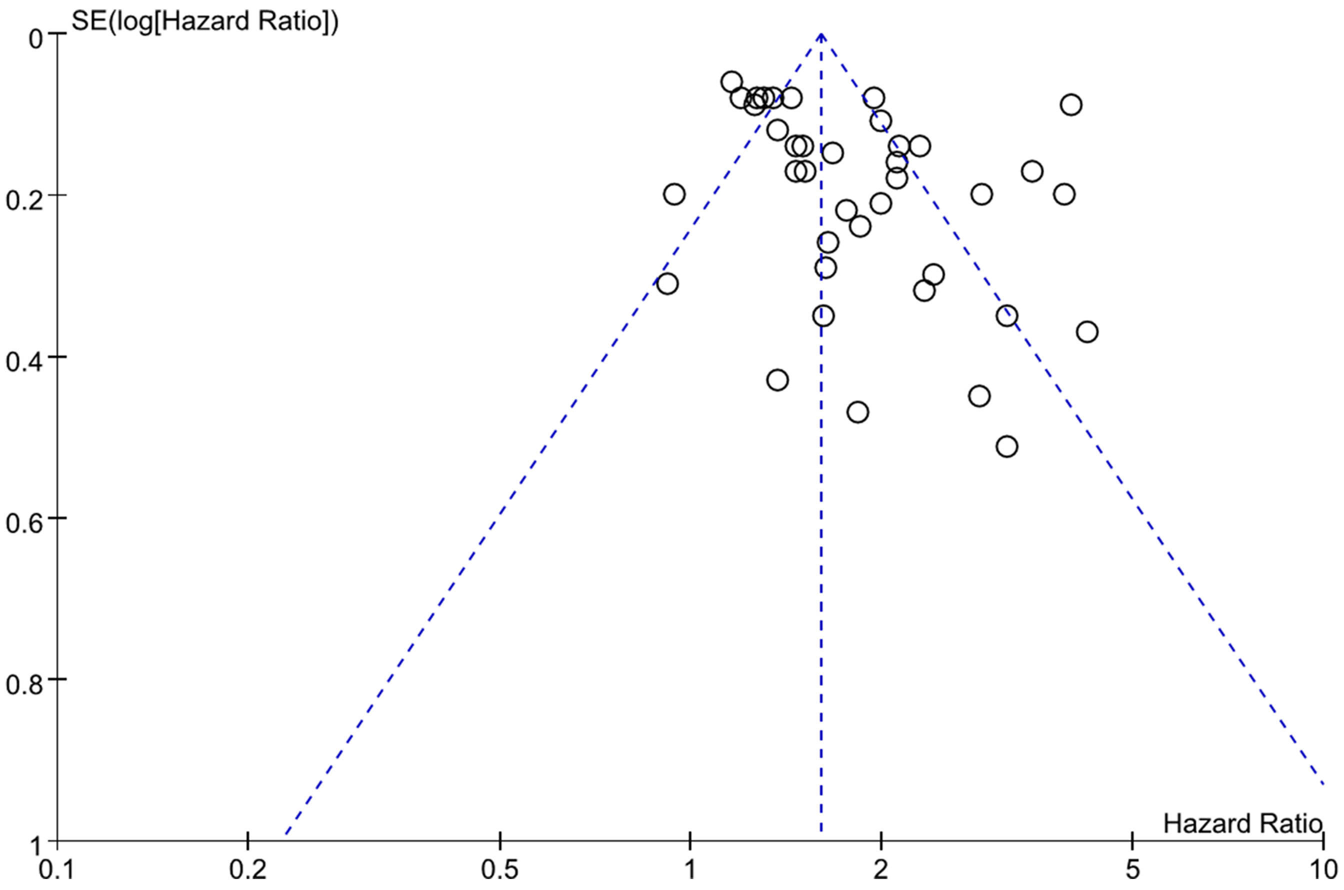

Supplementary Figure S1: Funnel plot regarding the association between baseline NLR and overall survival in HCC patients.

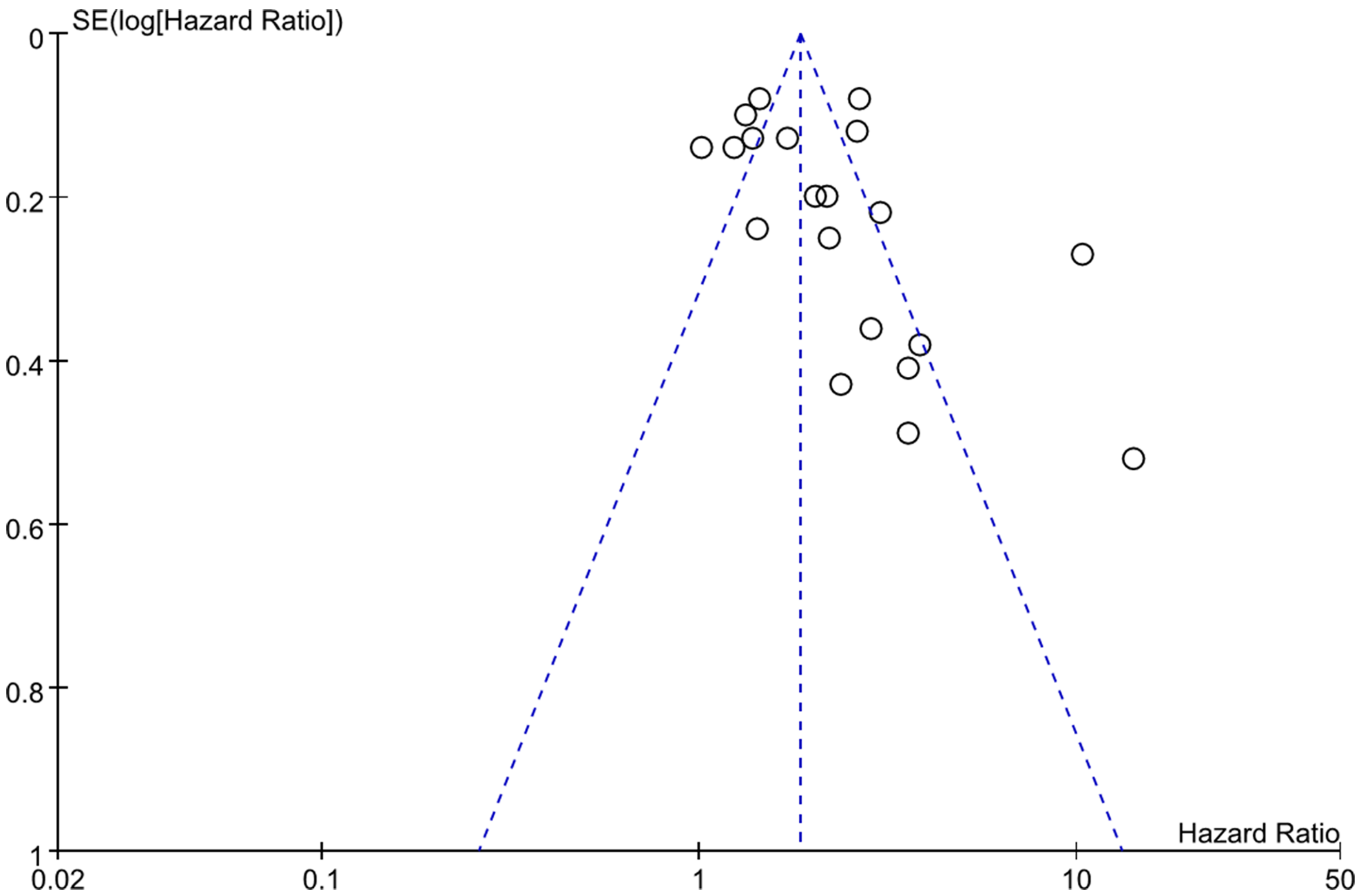

Supplementary Figure S2: Funnel plot regarding the association between baseline NLR and recurrence-free or disease-free survival in HCC patients.

Supplementary Table S1: Quality assessment of non-randomized studies using NEWCASTLE - OTTAWA QUALITY ASSESSMENT SCALE COHORT STUDIES

First Author, Journal (Year)	Q1	Q2	Q3	Q4	Q5	Q6	Q7	Q8	Total score
Abdelmessih RM	1 point	1 point	0 point	0 point	0 point	0 point	0 point	0 point	2 points
Afshar M	1 point	1 point	1 point	0 point	0 point	1 point	1 point	0 point	5 points
Agopian VG	1 point	1 point	0 point	0 point	0 point	0 point	0 point	0 point	2 points
Aino H	1 point	1 point	0 point	0 point	0 point	1 point	1 point	0 point	4 points
Bertuzzo VR	1 point	1 point	0 point	0 point	0 point	1 point	1 point	0 point	4 points
Bodzin A	1 point	1 point	0 point	0 point	0 point	0 point	1 point	0 point	3 points
Bronson N	1 point	1 point	0 point	0 point	0 point	1 point	1 point	0 point	4 points
Bruixola G	1 point	1 point	0 point	0 point	0 point	0 point	1 point	0 point	6 points
Chan AW	1 point	1 point	1 point	0 point	1 point	1 point	0 point	0 point	5 points
Chang JX	1 point	1 point	0 point	0 point	0 point	0 point	0 point	0 point	2 points
Chen TM	1 point	1 point	0 point	0 point	2 points	1 point	1 point	0 point	6 points
Chen X	1 point	1 point	0 point	0 point	0 point	0 point	1 point	1 point	6 points
Chen Z	0 point	1 point	0 point	0 point	0 point	0 point	0 point	0 point	1 point
da Fonseca LG	1 point	1 point	1 point	0 point	0 point	1 point	1 point	0 point	5 points
Dan J	1 point	1 point	0 point	0 point	2 points	1 point	1 point	0 point	6 points
Facciorusso A	1 point	1 point	0 point	0 point	0 point	1 point	1 point	0 point	4 points
Fan W	1 point	1 point	0 point	0 point	0 point	1 point	1 point	0 point	4 points
Fu SJ	1 point	1 point	0 point	0 point	1 point	1 point	1 point	0 point	5 points
Fu YP	0 point	1 point	0 point	0 point	0 point	0 point	1 point	0 point	2 points
Gao F	1 point	1 point	0 point	0 point	0 point	0 point	1 point	0 point	3 points
Gomez D; Farid S	1 point	1 point	0 point	0 point	1 point	1 point	1 point	1 point	6 points
Guo ZX	1 point	1 point	0 point	0 point	2 points	1 point	1 point	1 point	6 points
Halazun KJ	1 point	1 point	0 point	0 point	1 point	1 point	1 point	0 point	6 points
Harimoto N	1 point	1 point	0 point	0 point	0 point	1 point	1 point	0 point	4 points
Higashi T	1 point	1 point	0 point	0 point	0 point	1 point	1 point	0 point	4 points
Hu B	1 point	1 point	0 point	0 point	0 point	1 point	1 point	0 point	4 points
Huang GQ	1 point	1 point	1 point	0 point	1 point	1 point	1 point	0 point	6 points
Huang J	1 point	1 point	0 point	0 point	0 point	1 point	1 point	0 point	4 points
Huang ZL	1 point	1 point	0 point	0 point	2 points	1 point	1 point	0 point	6 points
Kanno Y	0 point	1 point	0 point	0 point	0 point	0 point	0 point	0 point	1 point
Kim DG	1 point	1 point	0 point	0 point	0 point	0 point	0 point	0 point	2 points
Kinoshita A	1 point	1 point	1 point	0 point	0 point	1 point	1 point	0 point	5 points
Lai Q	1 point	1 point	0 point	1 point	0 point	1 point	1 point	1 point	5 points
Li C	1 point	1 point	0 point	0 point	0 point	1 point	1 point	0 point	4 points
Li JP	1 point	1 point	0 point	0 point	0 point	1 point	1 point	1 point	5 points
Li X (Tumor Biology)	1 point	1 point	1 point	0 point	1 point	0 point	1 point	0 point	5 points
Li X (PLoS ONE)	1 point	1 point	0 point	0 point	1 point	1 point	1 point	0 point	5 points
Liao R	1 point	1 point	0 point	0 point	2 points	1 point	1 point	0 point	6 points
Liao W	1 point	1 point	0 point	0 point	0 point	1 point	1 point	0 point	4 points
Liese J	1 point	1 point	0 point	0 point	0 point	0 point	0 point	0 point	2 points
Limaye AR	1 point	1 point	0 point	0 point	2 points	1 point	1 point	0 point	6 points
Long J	1 point	1 point	0 point	0 point	0 point	1 point	1 point	0 point	4 points
Lu D	0 point	1 point	0 point	0 point	0 point	0 point	0 point	0 point	1 point
Luè A	1 point	1 point	0 point	0 point	0 point	0 point	0 point	0 point	2 points
Mano Y	1 point	1 point	0 point	0 point	0 point	1 point	1 point	0 point	4 points

McNally ME	1 point	1 point	0 point	0 point	2 points	1 point	1 point	0 point	6 points
Mizukoshi E	0 point	1 point	0 point	0 point	0 point	0 point	0 point	0 point	1 point
Motomura T	1 point	1 point	0 point	0 point	2 points	0 point	1 point	0 point	5 points
Na GH	1 point	1 point	0 point	0 point	2 points	1 point	1 point	0 point	6 points
Nagai S	0 point	1 point	1 point	0 point	0 point	0 point	0 point	0 point	2 points
Ni XC	1 point	1 point	0 point	0 point	2 points	0 point	0 point	0 point	4 points
Oh BS	1 point	1 point	0 point	0 point	0 point	0 point	1 point	0 point	3 points
Okamura Y	1 point	1 point	0 point	0 point	0 point	0 point	1 point	0 point	3 points
Parisi I	1 point	1 point	0 point	0 point	0 point	1 point	0 point	0 point	3 points
Peng W	1 point	1 point	0 point	0 point	2 points	1 point	1 point	1 point	7 points
Pinato DJ (TR)	0 point	1 point	0 point	0 point	0 point	0 point	1 point	0 point	2 points
Pinato DJ (JH)	1 point	1 point	0 point	0 point	0 point	0 point	1 point	0 point	3 points
Ruan DY	1 point	1 point	0 point	0 point	0 point	1 point	1 point	0 point	4 points
Shindoh J	1 point	1 point	0 point	0 point	0 point	1 point	0 point	0 point	3 points
Sirin G	1 point	1 point	0 point	0 point	0 point	0 point	0 point	0 point	2 points
Sukato DC	1 point	1 point	0 point	0 point	2 points	1 point	1 point	1 point	7 points
Sullivan KM	1 point	1 point	1 point	0 point	0 point	0 point	0 point	0 point	3 points
Sun Q	1 point	1 point	0 point	0 point	1 point	0 point	1 point	0 point	4 points
Tajiri K (HR)	1 point	1 point	0 point	0 point	0 point	1 point	1 point	0 point	4 points
Tajiri K (JGH)	1 point	1 point	0 point	0 point	0 point	1 point	1 point	0 point	4 points
Terashima T	1 point	1 point	1 point	0 point	1 point	1 point	1 point	0 point	6 points
Uchida K	1 point	1 point	0 point	0 point	0 point	0 point	1 point	0 point	3 points
Wang GY	1 point	1 point	0 point	0 point	0 point	1 point	1 point	0 point	4 points
Wang K	1 point	1 point	0 point	0 point	0 point	0 point	0 point	0 point	2 points
Wang Q	1 point	1 point	1 point	0 point	0 point	1 point	1 point	0 point	5 points
Wang W	1 point	1 point	0 point	0 point	0 point	1 point	1 point	0 point	4 points
Wei K	1 point	1 point	0 point	0 point	2 points	1 point	1 point	0 point	6 points
Weinmann AJ	1 point	1 point	0 point	0 point	0 point	0 point	0 point	0 point	2 points
Xiao GQ	1 point	1 point	1 point	0 point	1 point	1 point	1 point	0 point	6 points
Xu X	1 point	1 point	0 point	0 point	1 point	1 point	1 point	0 point	5 points
Xue TC	1 point	1 point	0 point	0 point	0 point	1 point	1 point	0 point	4 points
Yamamura K	1 point	1 point	0 point	0 point	0 point	1 point	1 point	0 point	4 points
Yang X	1 point	1 point	0 point	0 point	2 points	1 point	1 point	1 point	7 points
Yang Z	1 point	1 point	0 point	0 point	0 point	1 point	1 point	0 point	4 points
Yip V	0 point	1 point	0 point	0 point	0 point	0 point	1 point	0 point	2 points
Yoshizumi T (AR)	1 point	1 point	0 point	0 point	0 point	1 point	0 point	1 point	4 points
Yoshizumi T (TR)	1 point	1 point	0 point	0 point	0 point	1 point	0 point	1 point	4 points
Yoshizumi T (HR)	1 point	1 point	0 point	0 point	0 point	1 point	0 point	1 point	4 points
Young AL	1 point	1 point	0 point	0 point	0 point	1 point	1 point	0 point	4 points
Zhang J	1 point	1 point	0 point	0 point	0 point	0 point	1 point	0 point	3 points
Zhang W	1 point	1 point	0 point	0 point	1 point	1 point	1 point	0 point	4 points
Zheng YB (APJCP)	1 point	1 point	0 point	0 point	2 points	1 point	1 point	0 point	6 points
Zheng YB (CJIIT)	1 point	1 point	0 point	0 point	2 points	1 point	1 point	0 point	6 points
Zhou D (SR)	1 point	1 point	0 point	0 point	0 point	1 point	1 point	0 point	4 points
Zhou DS (WJG)	1 point	1 point	0 point	0 point	0 point	1 point	1 point	0 point	4 points
